# Immunomodulator FTY720 improves glucose homeostasis and diabetic complications by rejuvenation of *β*‐cell function in nonhuman primate model of diabetes

**DOI:** 10.1111/fcp.12760

**Published:** 2022-02-02

**Authors:** Yixin (Jim) Wang, Xiaoli Wang, Annie An, Mingfa Zang, Ling Xu, Kefeng Gong, Weihua Song, Qing Li, Xiaojun Lu, Yong‐Fu Xiao, Guoliang Yu, Zhongmin A. Ma

**Affiliations:** ^1^ Crown Bioscience Inc. San Diego California USA; ^2^ Innoland Bioscience Inc. Taicang China; ^3^ The First People's Hospital of Taicang Taicang Affiliated Hospital of Soochow University Suzhou China; ^4^ Apollomics Biopharmaceuticals, Inc. Hangzhou China; ^5^ Pegasus Bio LLC Great Neck New York USA

**Keywords:** cardiac dysfunction, CD8 + T lymphocytes, glucose tolerance, immunomodulation, insulin resistance, proteinuria, sphingosine 1‐phosphate (S1P), spontaneous diabetic nonhuman primate (NHP)

## Abstract

Inadequate *β*‐cell mass is essential for the pathogenesis of type 2 diabetes (T2D). Previous report showed that an immunomodulator FTY720, a sphingosine 1‐phosphate (S1P) receptor modulator, sustainably normalized hyperglycemia by stimulating *β*‐cell in vivo regeneration in *db/db* mice. We further examined the effects of FTY720 on glucose homeostasis and diabetic complications in a translational nonhuman primate (NHP) model of spontaneously developed diabetes. The male diabetic cynomolgus macaques of 18–19 year old were randomly divided into Vehicle (Purified water, *n* = 5) and FTY720 (5 mg/kg, *n* = 7) groups with oral gavage once daily for 10 weeks followed by 10 weeks drug free period. Compared with the Vehicle group, FTY720 effectively lowered HbA1c, blood concentrations of fasting glucose (FBG) and insulin, hence, decreased homeostatic model assessment of insulin resistance (HOMA‐IR); ameliorated glucose intolerance and restored glucose‐stimulated insulin release, indicating rejuvenation of *β*‐cell function in diabetic NHPs. Importantly, after withdrawal of FTY720, FBG, and HbA1c remained at low level in the drug free period. Echocardiography revealed that FTY720 significantly reduced proteinuria and improved cardiac left ventricular systolic function measured by increased ejection fraction and fractional shortening in the diabetic NHPs. Finally, flow cytometry analysis (FACS) detected that FTY720 significantly reduced CD4 + and CD8 + T lymphocytes as well as increased DC cells in the circulation. Immunomodulator FTY720 improves glucose homeostasis via rejuvenation of *β*‐cell function, which can be mediated by suppression of cytotoxic CD8 + T lymphocytes to *β*‐cells, thus, may be a novel immunotherapy to reverse T2D progression and ameliorate the diabetic complications.

**Abbreviations**
AUC: Area under the curve
BrdU: 5‐bromo‐2'deoxyuridine
Cmax: Maximum concentration
CO: Cardiac output
E/A ratio: Ratio of the left ventricular early over late trans‐mitral Doppler inflow velocity
EDV: Left ventricular end diastolic volume
EF/FS: Left ventricular ejection fraction/Fractional shortening
ESV: Left ventricular end systolic volume
FACS: Florescence activating cell sorter
FBG: Fasting blood glucose concentration
FTY720: (2‐amino‐2‐[4‐octylphenyl] ethyl)‐1,3‐propanediol; S1P, sphingosine 1‐phosphate
HDL: High density lipoprotein concentration
HOMA‐IR: Homeostatic Model Assessment of Insulin Resistance
LDL: Low density lipoprotein concentration
LN: Lymph note
LVIDd: Left ventricular inner diameter at diastole
NHP: Nonhuman primate
o/ivGTT: Oral/Intravenous glucose tolerance test
T_1/2_
: Terminal half life
T2D/DM: Type 2 diabetes/Diabetes Miletus
TC: Total cholesterol concentration
TG: Total triglyceride concentration
Tmax: The time to reach maximal concentration
Vz: The apparent volume of distribution during the terminal phase

## 1 BACKGROUND

Type 2 diabetes (T2D) is characterized by insulin resistance and reduction of functional pancreatic *β*‐cell mass [1, 2]. Although there is an initial compensatory increase in *β*‐cell mass against insulin resistance in early stage of the disease progression, diabetes occurs when the functional *β*‐cell mass fails to expand sufficiently [3, 4]. Current treatments for T2D are only able to ameliorate diabetes symptoms by decreasing hyperglycemia without halting the causes of the disease. Development of a strategy to restore the mass of functional *β*‐cells in diabetic patients is therefore a key step for the cure/reverse of T2D in humans [2, 5, 6, 7, 8].

Increasing evidence suggests that immune system plays an essential role in the progression of T2D [9, 10]. A resent Nature paper revealed that autoimmune stem‐like CD8 + T cells destroy the *β*‐cells to cause T1D [11]. FTY720 (Gilenya, Fingolimod) was developed as an immunosuppressant to treat multiple sclerosis with the mechanism of action that FTY720 downregulates sphingosine 1‐phosphase 1 (S1P1) receptor on lymphocytes, thereby prevents lymphocyte egress from lymphoid tissues, which, in turn, reduces the infiltration of auto‐aggressive cells into the central nervous system [12, 13]. Researchers found that FTY720 also showed effect to prevent autoimmune diabetes (T1D) in nonobese diabetic (NOD) mice [14] as well as in *db/db* mice (T2D) leading to a sustained normalization of hyperglycemia by stimulating in vivo regeneration of *β*‐cells [15]. Importantly, normalized blood glucose can be maintained even after withdrawal of FTY720 treatment, indicating that immunotherapy could be a curative approach for the treatment of T2D [15]. The evidence of FTY720 induced *β*‐cell regeneration in the islets from *db/db* mice includes: (1) increasing *β*‐cell proliferation through a PI3K‐dependent downregulation of p57^KIP2^ and upregulation cyclin D3; (2) increasing the neogenesis of *β*‐cells from pancreatic duct region through upregulation of PDX‐1 expression; and (3) increasing *β*‐cell survival by upregulation of Bcl‐2 and BcL‐xL [15, 16].

With the limitations of the rodent models of diabetes, from which the experiment results often are unable to translate to human diseases, the present study aimed to further explore the therapeutic potential of FTY720 in the treatment of T2D using a nonhuman primate (NHP) model with spontaneously developed diabetes, which has been proved to be highly resemble all the characteristics of T2D in human patients at different stages of the disease progression, and is widely used in academia and pharmaceutical industry as by far the most predictive animal model for both the basic and preclinical research in testing novel therapeutics for human metabolic diseases, including diabetes [17, 18, 19, 20, 21, 22]. Interestingly, the spontaneous diabetes NHPs clearly showed *β*‐cell failure in response to glucose stimulation [20], thus, the findings in this well‐characterized naturally occurred diabetic model can provide significant insight information for development of FTY720 as a therapeutic drug for the treatment of T2D in human patients.

## 2 METHODS

FTY720 (2‐amino‐2‐[4‐octylphenyl] ethyl)‐1,3‐propanediol was purchased from Nanjing Chemipioneer Pharma & Tech Co., Ltd., (China). Dosing solution was prepared weekly in purified water at a concentration of 5 mg/ml and stored at 4°C for daily oral administration at a volume of 1 ml/kg and 5 mg/kg.

### 2.1 Animals and procedures

The male cynomolgus macaques (*Macaca fascicularis*) were individually housed in species appropriate cages at temperature‐controlled rooms (20 ± 3°C) on a 12‐h light–dark (6:00–18:00) cycle, were fed normal primate chow containing 19% protein, 5% fat and 3.6% fiber (Shanghai Shilin Biotechnology Inc., Shanghai, China) twice daily and had free access to tap water. All animal procedures used in this study were in accordance with the guidelines of the Association for Assessment and Accreditation of Laboratory Animal Care (AAALAC) and approved by the Crown Bioscience institutional animal care and use committee (IACUC).

After 2‐week acclimation and training, the baseline blood chemistries, including fasting blood glucose (FBG), hemoglobin A1c (HbA1c), blood cell count (CBC), and so on were measured, based on which, qualified NHPs were enrolled and randomly divided into two groups: Vehicle (*n* = 6) and FTY720 (*n* = 7) with once daily oral gavage of purified water (1 ml/kg) or FTY720 (5 mg/kg), respectively. After 10‐week initial treatment, two original groups were switched each other, namely, the daily treatment in the original vehicle group was changed to FTY720 and in the original FTY720 group changed to the purified water for continuous observation for another 10 weeks as a washout period. During the 10 weeks treatment, the physical condition of one NHP in the Vehicle group was not stable, which was then excluded from the study, thus, only five NHPs were included in the efficacy study. All NHPs were subjected to continuously monitoring food intake (FI), body weight (BW), the blood concentrations of fasting glucose and insulin, HbA1c, and so on every 2 weeks. The systemic insulin resistance index was calculated based on homeostatic model assessment for insulin resistance: HOMA‐IR = Insulin (mIU/L) × glucose (mmol/L) /22.5 [23].

### 2.2 Pharmacokinetics (PK) and pharmacodynamics (PD) of FTY720 in the diabetic nonhuman primates

Blood samples were taken in seven conscious diabetic NHPs immediately before (0 h), and at 0.5, 1, 2, 4, 6 h after the first dose and 1, 2, 7, and 14 days and then every 2 weeks immediately before the following daily repeated dose of FTY720 (5 mg/kg) for 10 weeks, as well as in the washout period for 10 more weeks.

#### 2.2.1 Pharmacokinetics (PK)

LC–MS/MS technology was used to measure blood concentrations of FTY720 with detailed analytic methodology described in the Supplement section. The plasma concentrations of FTY720 at each time point in 0–24 h after the 1st dose of FTY720 from each individual NHP were used for calculation of the PK parameters by linear trapezoidal interpolation and Lambda Z best fit under non‐compartmental model (WinNonlin 8.2). When less than three points were available after peak plasma concentration (Cmax) was established, the best fit model cannot calculate the terminal half‐life (t_1/2_) value.

#### 2.2.2 Pharmacodynamics (PD)

The counts of blood cell (CBC) was used as a PD biomarker to measure the systemic functional kinetics of FTY720.

### 2.3 Glucose tolerance test (GTT)

#### 2.3.1 Intravenous glucose tolerance test (ivGTT) in anesthetized NHPs

After treatment of either vehicle (*n* = 5) or FTY720 (*n* = 7) for 10 weeks, the diabetic NHPs were fasted for 16 h and then anesthetized with intramuscular injection of ketamine (16 mg/kg), supplemented with 0.16 mg/kg as needed. Blood samples were collected prior (0 min) and post (3, 5, 7, 10, 15, 20, and 30 min) iv administration of Dextrose (50%, 250 mg/kg) via saphenous vein for measurements of blood concentrations of glucose, insulin, and C‐peptide by SIEMENS ADVIA‐2400 [24, 25].

#### 2.3.2 Oral glucose tolerance test (oGTT) in conscious NHPs

In a separate experiment, oGTT was performed before (baseline) and after treatment of FTY720 for 8 weeks in the same 12 diabetic NHPs. On the experiment day, following 16 h fasting, blood glucose concentrations were measured by a Glucometer (Roche, Accu‐chek) prior (0 min) and post (30, 60, 90, 120, and 180 min) oral glucose loading via gavage (35%, 1.75 g or 5 ml/kg body weight) [26].

### 2.4 Florescence activating cell sorter analysis

About 24 h after the last dosing, circulating blood samples were collected from two groups of diabetic (DM) NHPs treated with once daily oral dose of vehicle (purified water, *n* = 6) or FTY720 (5 mg/kg, *n* = 7) and a group of normal NHPs treated with vehicle as control (Normal + Veh, *n* = 7) for 8 weeks. The blood samples were lysed by RBC lysis and stained according to the user manual (BD Bioscience) for all antibodies against CD3 PerCP‐cy5.5, CD4 BV605, CD8 PE, CD11c BV650, CD56 PE‐cy7, CD127 AF647, and CCR6 FITC, respectively. The immune cells were analyzed by a flow cytometry (Fortessa) with the gating strategy described in the supporting information.

### 2.5 Statistical analysis

Data were expressed as mean value ± standard error of the mean (SEM). Significant differences among groups were evaluated by one‐way ANOVA and Turkey's multiple comparison test or by unpaired two‐tailed Student's *t* test using a statistic software (PRISM). Significant levels of the difference among the comparison groups are set as *p* value < 0.05.

## 3 RESULTS

### 3.1 Pharmacokinetics (PK) and pharmacodynamics (PD) of FTY720 in NHPs (Figure 1)

**FIGURE 1:**
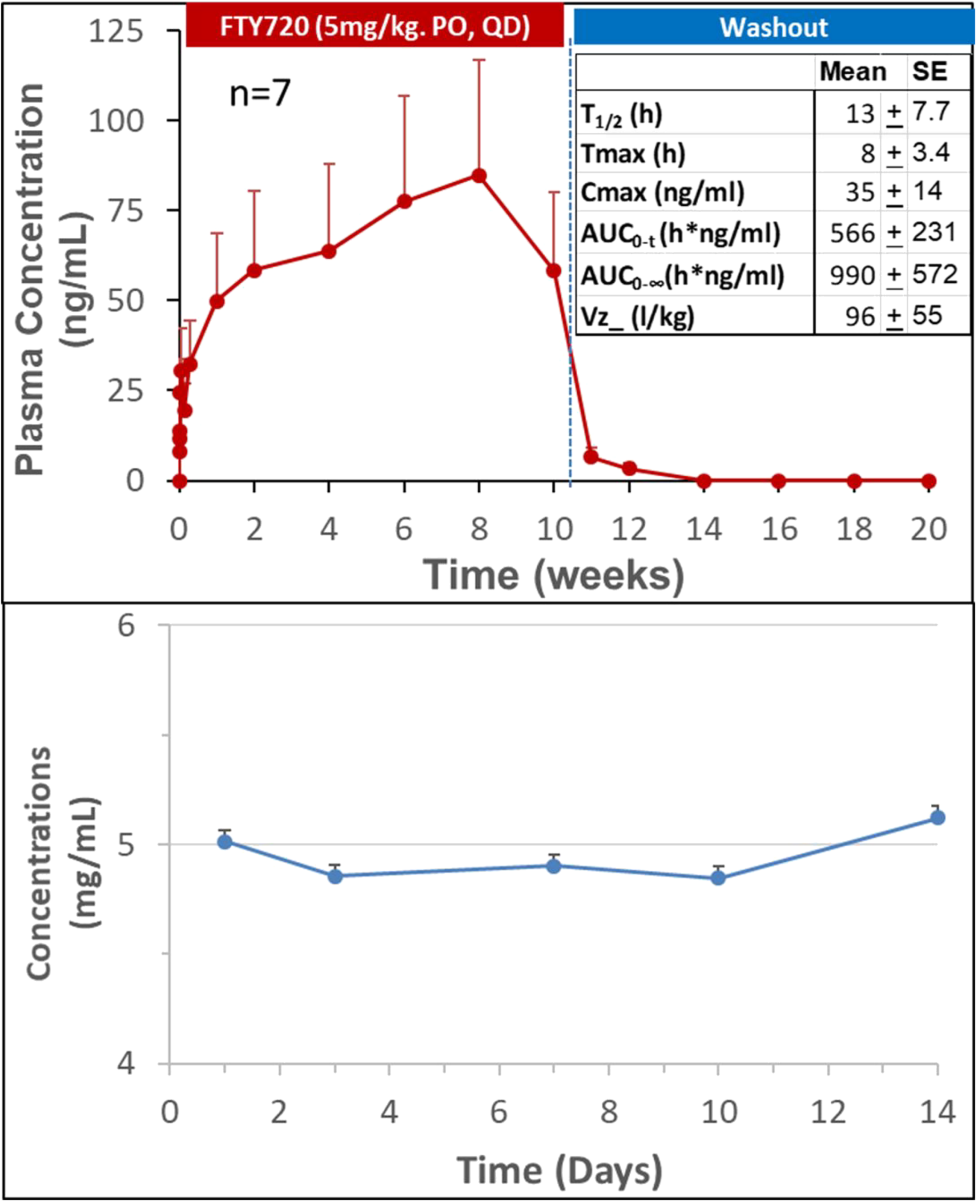
Pharmacokinetics of FTY720 in the diabetic nonhuman primates (NHPs) (PK). Top: Plasma concentrations of FTY720 after daily oral administration (5 mg/kg) for 10 weeks followed by 10‐week washout, measured at time 0 (Baseline), and 0.5, 1, 2, 4, 8, and 24 h after the first dose to calculate PK profile, and at day 2, 7, and 14, every 2 weeks thereafter following repeated daily dose, and then, termination of FTY720 administration, respectively. Bottom: Stability of FTY720 in dosing solution at 4°C for 2 weeks

The PK and cell trafficking dynamics of FTY720 in normal cynomolgus monkeys has been reported previously [27]. In the present study, the PK and PD characteristics of FTY720 was examined in the spontaneous diabetic NHPs.

#### 3.1.1 Pharmacokinetics (PK)

Following the first oral dose of FTY720 (5 mg/kg), plasma concentration of FTY720 gradually elevated, reached to peak 35 ng/ml (Cmax) at ∼ 8 h (Tmax) with a terminal half‐life (*t*_1/2_) ∼ 17 h (Top). Thereafter, the blood drug concentrations accumulatively increased following repeated daily dose of FTY720; and then quickly diminished to 7 and 3 ng/ml on the 1st and 2nd week, respectively; and to below detectable levels in the remaining washout period when the drug administration was stopped. The stability of FTY720 in the dosing solution (5 mg/ml) was measured at day 3, 7, 10, and 14 with a recovery rate ranging from 95–104% (Bottom).

#### 3.1.2 Pharmacodynamics (PD)

The counts of blood cell (CBC), used as a PD biomarker to measure the systemic functional kinetics of FTY720, showed that lymphocytes were lowered significantly by about ∼50%, while total white blood cells and neutrophils were moderately reduced (Table 1). Apparently, FTY720 treatment for 10 weeks had no significant effects on body weight, BMI, blood concentrations of lipids and C‐reactive protein (Table 1).

**TABLE 1:** General characteristics of the diabetic NHPs following once daily oral administration of vehicle (purified water, 1 ml/kg) or FTY720 (5 mg/kg) for 10 weeks

Group	Vehicle	FTY720	*t* test
Number of Animals	6	7	*p* value
**General**			
Age (years)	18 ± 1.2	19 ± 0.9	0.79
Body weight (kg)	8.1 ± 1.3	8.0 ± 0.9	0.97
BMI (kg/m2)	13 ± 1.4	13 ± 0.9	0.74
**Blood Chemistry**			
TC (mmol/L)	3.9 ± 0.4	3.4 ± 0.3	0.30
TG (mmol/L)	2.9 ± 0.5	3.4 ± 0.8	0.69
HDL (mmol/L)	1.1 ± 0.1	0.8 ± 0.1	0.16
LDL (mmol/L)	1.7 ± 0.3	1.3 ± 0.1	0.26
C‐Reactive Protein (mg/L)	2.4 ± 0.3	2.6 ± 0.3	0.61
**Blood cells**			
White blood Cells (109/L)	11 ± 1.0	7.9 ± 1.1	0.06
Neutrophil (109/L)	4.8 ± 0.8	4.2 ± 0.6	0.56
Lymphocyte (109/L)	5.4 ± 0.5	2.7 ± 0.5	**0.00**

*Note*: *P* value is calculated by *t* test between the two groups and the statistically significant difference was highlighted in bold when p value is less than 0.05.

### 3.2 FTY720 reduced glycemia and insulin resistance (Figure 2)

**FIGURE 2:**
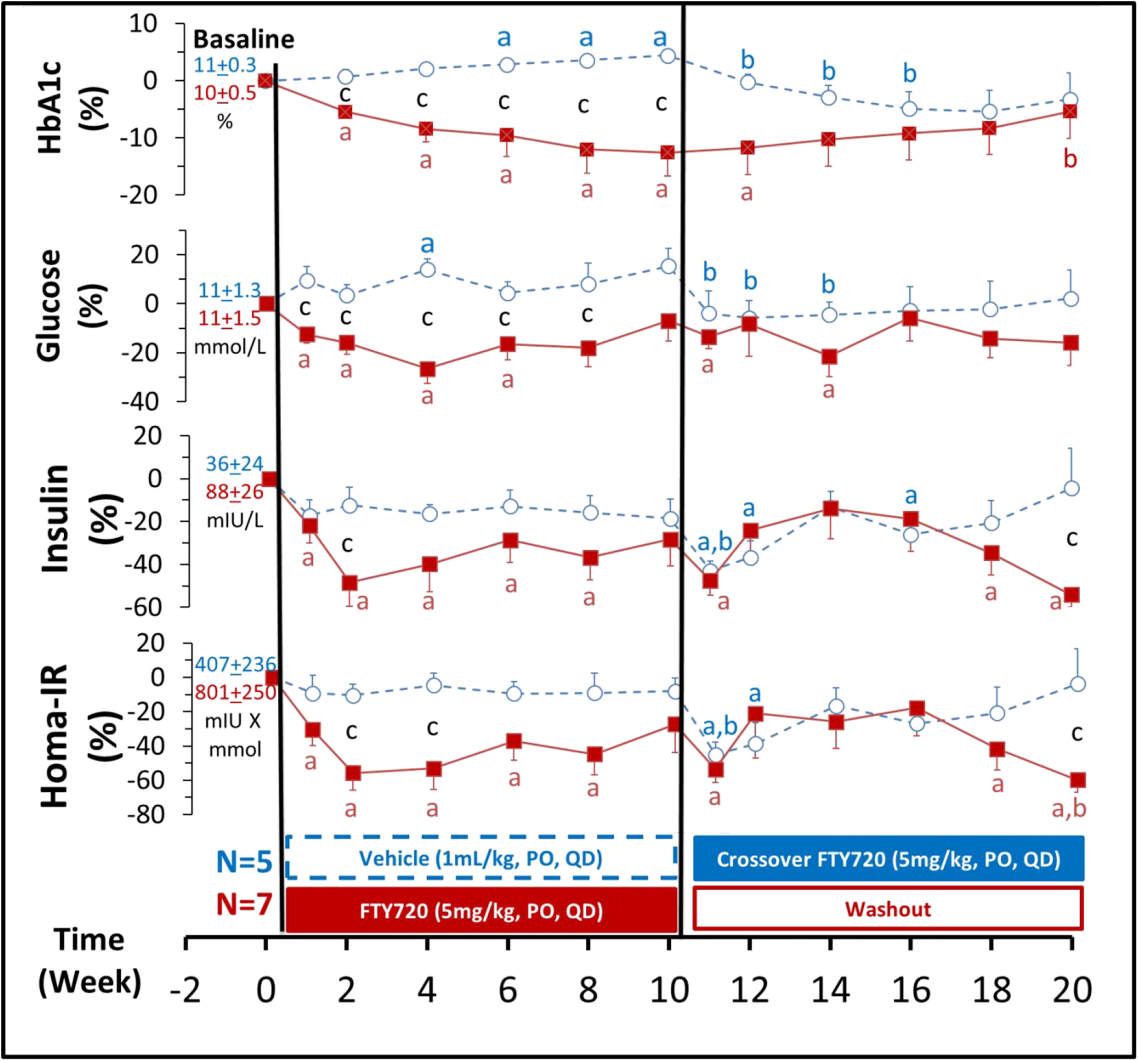
FTY720 decreased glycemia and insulin resistance. From top to bottom: Percent changes of blood hemoglobin A1c (HbA1c), glucose and insulin concentrations, and calculated Homeostatic Model Assessment of Insulin Resistance Index (Homa‐IR) from baseline (week 0) and after once daily oral administration of vehicle (purified water, 1 ml/kg, *n* = 5) or FTY720 (5 mg/kg, *n* = 7) for 10 weeks in the diabetic nonhuman primates (NHPs). The actual baseline values are presented next to the first time point colored blue as vehicle and red as FTY720 group, respectively. After week 10, the treatments were crossover, the original control group started with vehicle administration (blue) was changed to FTY720 (5 mg/kg, PO. QD); while the drug administration in the original FTY720 group (red) was stopped for continue observation as a washout period in the next 10 weeks. Statistical significance: *p* value < 0.05: Paired *t* test: a. Post‐ versus pre‐dosing value at week 0 (baseline) in the same NHP. b. Post‐ versus pre‐crossover value at week 10 in the same NHP. Unpaired *t* test: c. Comparison of the values between the vehicle and FTY720 group at each corresponding time points

To evaluate the effect of FTY720 treatment on glycemic homeostasis in the diabetic NHP, HbA1c, blood concentrations of fasting glucose (FBG) and insulin were measured biweekly. Both HbA1c and FBG were significantly reduced and remained at low levels with a slow rise during washout period after termination of FTY720 treatment. In contrast in the control group of diabetic NHPs, both HbA1c and FBG were gradually drifting up during vehicle treatment period. Interestingly, the increased FBG and HbA1c levels were significantly decreased during the crossover period when the treatment changed from the vehicle to FTY720 at week 10.

Following the decrease in glycemia, blood concentrations of insulin also reduced in the diabetic NHPs treated with FTY720, in parallel, the calculated insulin resistance indices (HOMA‐IR) decreased, indicating improvement of tissue insulin sensitivity.

### 3.3 FTY720 restored glucose‐stimulated insulin response and ameliorated glucose intolerance (Figure 3)

**FIGURE 3:**
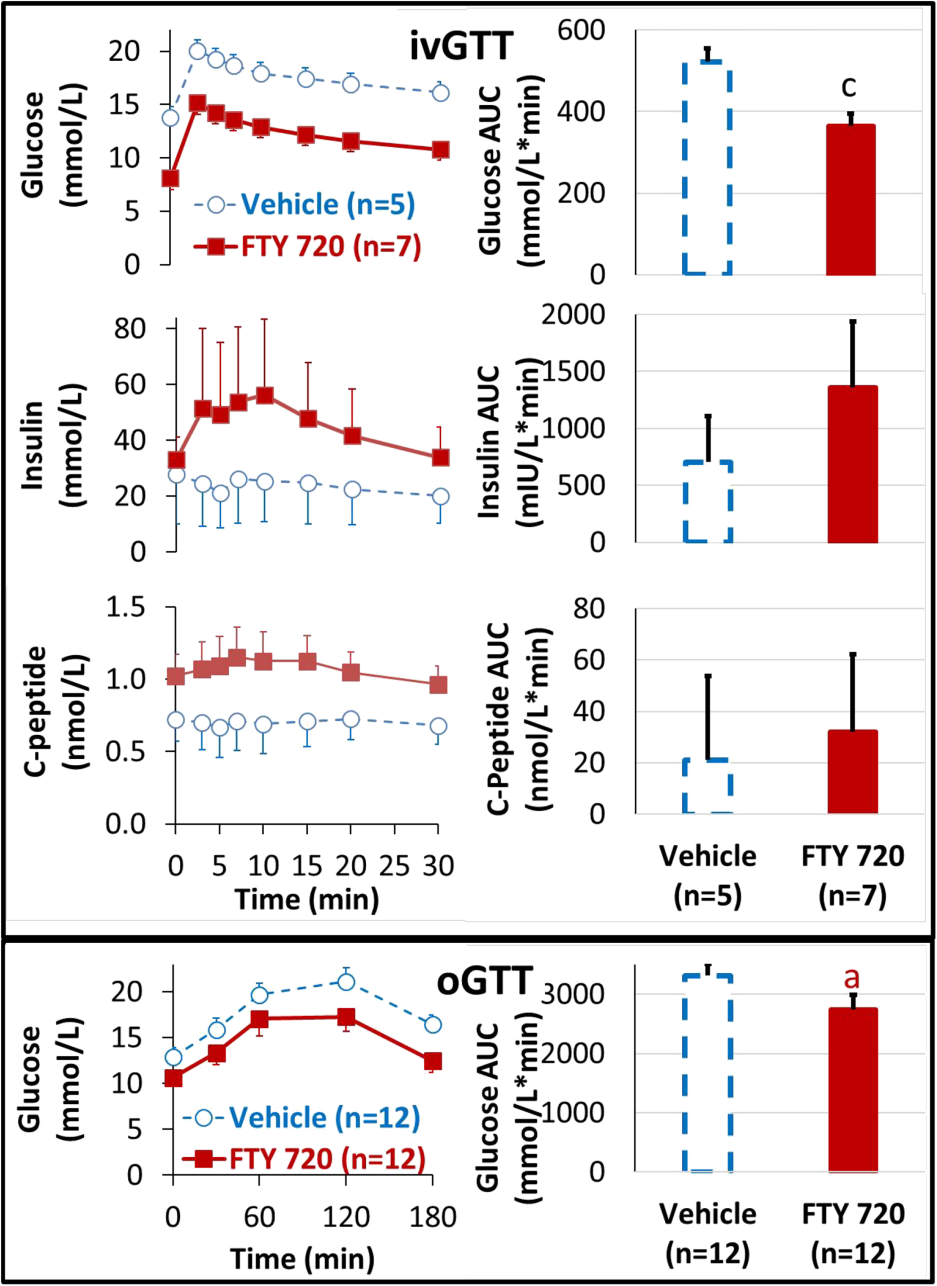
FTY720 restored glucose‐stimulated insulin response (ivGTT) and ameliorated glucose intolerance (oGTT). Top: Intravenous glucose tolerance test (ivGTT) in anesthetized diabetic nonhuman primates (NHPs) after once daily oral administration of vehicle (blue, purified water 1 ml/kg, *n* = 5) or FTY720 (red, 5 mg/kg, *n* = 7) for 10 weeks. Concentrations (left) and integration of the area under the concentration curve (AUC, right) of blood glucose, insulin, and C‐peptide. “c”: *p* < 0.05, unpaired *t* test, vehicle versus FTY720 group at week 10 after respective treatment. Bottom: In a separate experiment, oral glucose tolerance test (oGTT) was conducted before (blue, vehicle) and after once daily oral administration of FTY720 (5 mg/kg) for 8 weeks (red, FTY720) in the same conscious diabetic NHPs (*n* = 12). “a”: *p* < 0.05, paired *t* test, baseline at week 0 (Vehicle) versus post‐treatment at week 8 (FTY720) in the same 12 NHPs

It was reported that administration of FTY720 to *db/db* mice led to sustained normalization of hyperglycemia by stimulating *β*‐cell in vivo regeneration without affecting the insulin sensitivity.^15^ Since the experimental NHP cannot be sacrificed to examine the morphological evidence of *β*‐cell regeneration, glucose tolerance test (GTT) was performed in anesthetized diabetic NHPs to evaluate *β*‐cell function with treatment of vehicle or FTY720.

#### 3.3.1 Intravenous glucose tolerance test (ivGTT) in anesthetized NHPs (top)

Following intravenous administration of glucose in the diabetic NHPs with overnight fasting (∼16 h), the blood glucose concentrations (baseline at time 0) was significantly higher, but both glucose‐stimulated insulin and C‐peptide responses were flat in the control diabetic NHPs treated with vehicle, indicating diminished *β*‐cell function to produce insulin. In contrast in the diabetic NHPs with daily administration of FTY720 (5 mg/kg) for 10 weeks, the glycemic response was significantly reduced measured by both the time course of glucose concentration curve (Left) and integration of the area under the glucose response curve (AUC, Right), while glucose‐stimulated insulin and C‐peptide responses were enhanced compared with the responses in the Vehicle group, indicating functional restoration of pancreatic *β*‐cells by FTY720.

#### 3.3.2 Oral glucose tolerance test (oGTT) in conscious NHPs (bottom)

The oGTT was performed in all 12 NHPs before and after FTY720 treatment for 8 weeks. On the experiment day, the NHPs were fasted for 16 h. Following oral glucose loading, blood glucose concentrations gradually elevated, however, the glycemic response, measured by both glucose concentration time course (Left) and AUC (Right), was significantly reduced in the second oGTT after FTY720 treatment for 8 weeks compared to the pre‐treatment baseline, indicating that FTY720 ameliorated glucose intolerance in the diabetic NHPs.

### 3.4 FTY720 enhanced cardiac left ventricular (LV) systolic functions (Figure 4)

**FIGURE 4:**
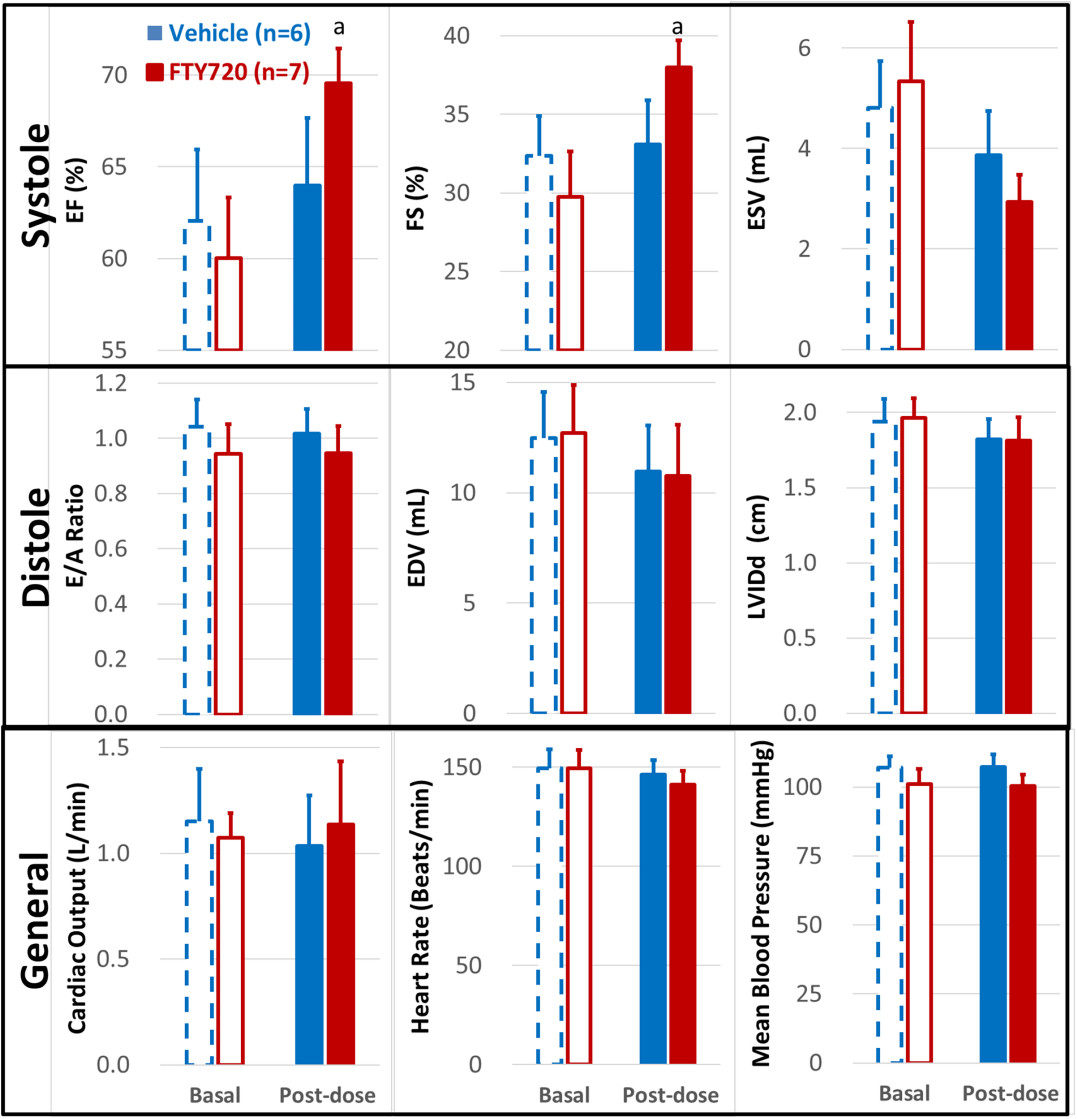
FTY720 enhanced left ventricular systolic function of the heart. Cardiac functions was measured noninvasively by echocardiography in anesthetized diabetic nonhuman primates (NHPs) before (basal) and 10 weeks after (post‐dose) once daily oral administration of vehicle (blue, *n* = 5) or FTY720 (red, 5 mg/kg, *n* = 7), which are grouped from top to bottom as below: *Systole*: Left ventricular ejection fraction (EF); fractional shortening (FS) and end systolic volume (ESV). *Diastole*: Ratio of left ventricular early over late trans‐mitral Doppler inflow velocity (E/A), end diastolic volume (EDV); and inter diameter at diastole (LVIDd). *General*: Cardiac output (CO); heart rate (HR); and mean arterial blood pressure (MBP). Statistical significance: *p* value < 0.05, a. paired *t* test, baseline versus post‐dose treatment of vehicle or FTY720 for 10 weeks in the same NHPs

The echocardiography revealed that the ejection fraction (EF) is significantly reduced from 68% in the normal NHPs to 62% in the diabetic NHPs and fractional shortening (FS) from 43% to 30% [17, 21]. In the present study, the echocardiography was performed in the anesthetized diabetic NHPs before (baseline) and 8 weeks after administration of the vehicle or FTY720. As shown in Figure 4, comparing to the baseline, FTY720 treatment significantly enhanced the LV systolic function measured by elevation of EF from 60% to 69% and FS from 30% to 38%, and moderately reduced the end systolic volume (ESV), which was slightly increased in the diabetic NHPs [17]. In addition, FTY720 treatment showed no significant effects on the LV diastolic function, including the E/A ratio, end diastolic volume (EDV) and LV inner diameter at diastole (LVIDd), nor on general cardiac function, including the cardiac output (CO), heart rate (HR) and mean blood pressure (MBP). These results suggest that FTY720 treatment significantly improved cardiac systolic function in the diabetic NHPs.

### 3.5 FTY720 diminished glucosuria and proteinuria (Figure 5)

**FIGURE 5:**
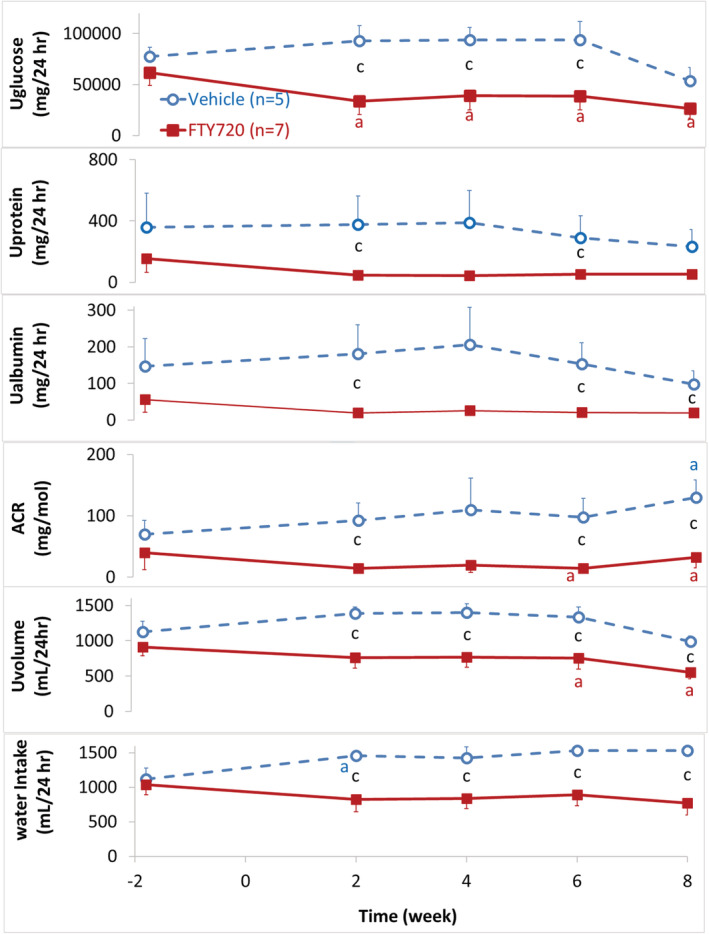
FTY720 diminished glucosuria and proteinuria. From top to bottom: Daily urinary excretion of glucose (Uglucose), protein (Uprotein), albumin (Ualbumin), albumin to creatinine ratio (ACR), volume (Uvolume) and water intake before (Week −2) and after once daily oral administration of vehicle (blue, purified water 1 ml/kg, *n* = 5) or FTY720 (red, 5 mg/kg, *n* = 7) in the diabetic nonhuman primates (NHPs). Statistical significance: *p* value < 0.05: “a”. Paired *t* test, Post‐ versus pre‐dosing value (baseline) at week −2 in the same NHP; “c”. Unpaired *t* test, comparison of the values between the vehicle and FTY720 group at each time points

Daily urinary excretion of glucose (Uglucose), protein (Uprotein), albumin (Ualbumin), albumin to creatinine ratio (ACR), urine volume (Uvolume) and water intake were measured 2 weeks before (baseline) and 8 weeks after once daily oral administration of vehicle (*n* = 5) or FTY720 (5 mg/kg, *n* = 7) in the diabetic NHPs. Over the experiment period, the urinary excretion of glucose, albumin and ACR, as well as the water intake gradually elevated, reaching significance from the baseline levels (week −2) at week 8 for ACR and week 2 for water intake in the diabetic NHPs treated with vehicle only, while in the FTY720 group, most of the urine parameters gradually decreased, reaching statistical significance for glucose at all time points, urine volume and ACR at weeks 6 and 8, suggesting that FTY720 treatment improved renal functions in the diabetic NHPs.

### 3.6 FTY720 decreased food intake without significantly impacting body weight (Figure 6)

**FIGURE 6:**
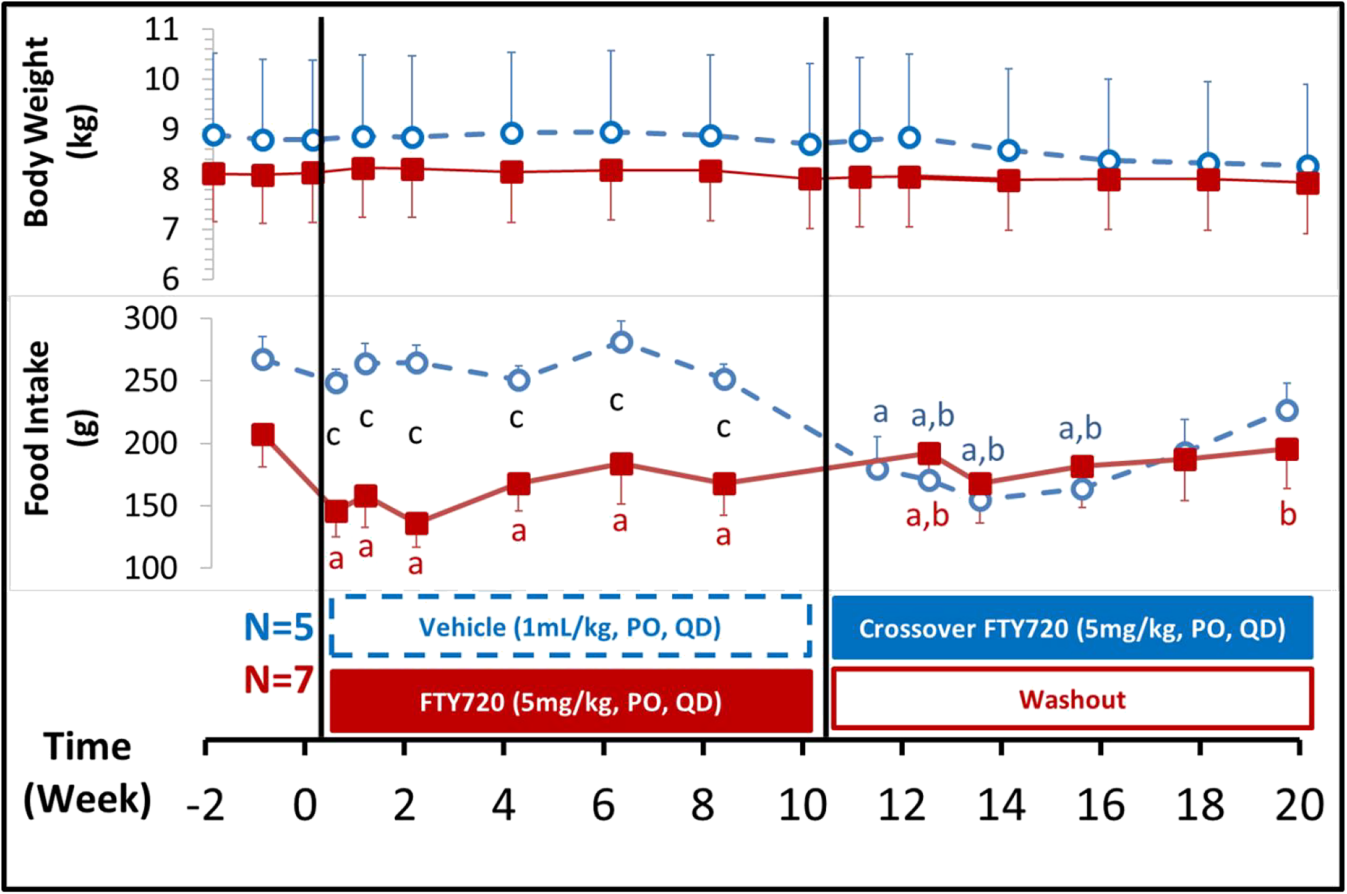
FTY720 decreased food intake without significantly impacting body weight. Body weight (top) and daily food intake before (Week −1) and after once daily oral administration of vehicle (blue, purified water 1 ml/kg, *n* = 5) or FTY720 (red, 5 mg/kg, *n* = 7) in the diabetic nonhuman primates (NHPs). Statistical significance: *p* value < 0.05: Paired *t* test for “a”. Post‐ versus pre‐dosing value (baseline) at week 0 in the same NHP; “b”. Post versus pre‐crossover value at week 10 in the same NHP; Unpaired *t* test: “c”. Compared the values between the vehicle and FTY720 group at each corresponding time points

Body weight and daily food intake were measured before and after once daily oral administration of vehicle (*n* = 5) or FTY720 at 5 mg/kg (*n* = 7) in the diabetic NHPs. Both the body weight and food intake were stable in the vehicle group. However, treatment of FTY720 significantly decreased food intake without affecting body weight. After stop dosing, the food intake slowly came back. Interestingly, the crossover treatment switching from the vehicle to FTY720 in the original control group reproducibly resulted in a similar decrease in food intake without apparent change in body weight.

### 3.7 FTY720 modulated peripheral immune‐cell profiles (Figure 7)

**FIGURE 7:**
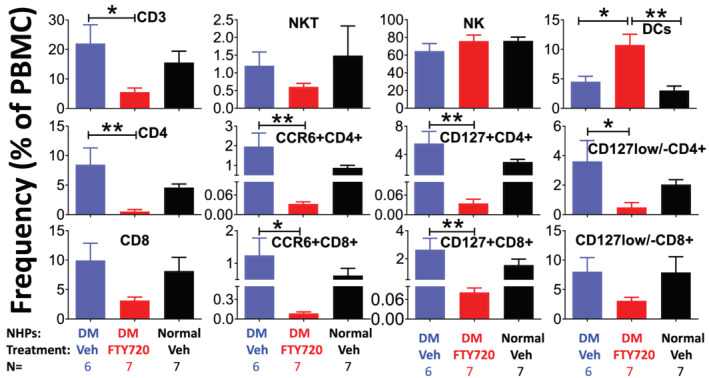
FTY720 modulated peripheral immune cell profiles. Percentage of individual positively stained live cells over total circulating peripheral blood mononuclear cells (PBMC), including T lymphocytes of CD3 + (total), CD4 + (effector), CD127 + (memory), CCR6 + (antigen experienced memory), CD8+, Natural Killer (NK), and dendritic cells (DC) in the diabetic nonhuman primates (NHPs) treated with once daily oral dose of the vehicle (Blue, purified water 1 ml/kg, DM + Veh, *n* = 6) or FTY720 (Red, 5 mg/kg, DM + FTY720, *n* = 7) and non‐diabetic NHPs treated with the vehicle (Black, Normal + Veh, *n* = 7) for 8 weeks. Blood samples were taken 24 h after the last dose for flow cytometry analysis

To analyze the peripheral immune cell profiles, the flow cytometry analysis was performed in the diabetic NHPs with once daily oral treatment of vehicle and FTY720 (5 mg/kg) for 10 weeks as well as a group of normal NHPs treated with the vehicle only. There were no significant differences for all analyzed cell lineages including CD3+, CD4+, and CD8 + T lymphocytes, as well as NKT, NK and DC cells between the diabetic and normal control NHPs treated with the vehicle. However, FTY720 treatment in the diabetic NHPs significantly increased the ratio of DCs and lowered the ratio of T lymphocytes, including CD3+, CD4+, CD8+, CD127+, and CCR6 + in the circulation, with no significant effects on NKT and NK cells compared to the diabetic NHPs treated with vehicle, indicating that FTY720 selectively modulated peripheral immune‐cell profiling.

## 4 DISCUSSION

We report here that oral administration of an immunomodulator FTY720 in spontaneous diabetic NHPs effectively lowered the FBG and HbA1c, which, intriguingly, remained at lower levels even after withdrawal of FTY720 treatment. This observation is consistent with the previous finding in *db/db* mice that FTY720 led to sustained normalization of hyperglycemia, which was also remained for life even after withdrawal of FTY20 [15]. The animal model used in the present study is well‐characterized naturally occurred diabetic NHPs with all the T2D characteristics in human patients at different stages of the disease progression [17, 18, 19, 20, 21, 22], particularly *β*‐cell failure [20]. To our knowledge, FTY720 is the only compound reported so far with a sustained anti‐glycemic effect even after cease of the treatment in both the diabetic mice and NHPs. These results suggest that treatment of T2D with FTY720 may be a novel immunotherapy that can potentially reverse T2D progression.

T2D is characterized by insulin resistance and reduction in functional pancreatic *β*‐cell mass [1, 2]. Although there is an initial compensatory increase of *β*‐cell mass in response to insulin resistance in early stage, diabetes occurs when the functional *β*‐cell mass fails to expand sufficiently in late stage of the disease [3, 4]. Indeed, in the present experiment, the glucose‐stimulated insulin and C‐peptide response during ivGTT was almost flat in the vehicle treated diabetic NHPs, indicating diminished *β*‐cell function, while FTY720 treatment restored *β*‐cell function evidenced by elevation in insulin and C‐peptide in response to glucose stimulation. It is known that FTY720 neither increases insulin sensitivity nor directly stimulates insulin secretion [15], therefore, restoration of *β*‐cell function in the FTY720‐treated group could be attributable to the increased functional *β*‐cell mass although we cannot sacrifice the diabetic NHP to obtain a direct evidence of *β*‐cell regeneration. Thus, it is proposed that development of a therapeutic strategy to increase the functional *β*‐cell mass in diabetic patients may lead to the cure/reverse of T2D in humans [2, 5, 6, 7, 8]. The present data from NHPs along with previous finding from *db/db* mice [15] strongly support this hypothesis.

Adult pancreatic *β*‐cells have been found to be mainly expanded by self‐duplication [28]. Interestingly, it was reported that FTY720 was able to stimulate functional *β*‐cell expansion in *db/db* mice mainly by increasing *β*‐cell proliferation through a PI3K‐dependent downregulation of the cell cycle inhibitor p57^KIP2^ and upregulation of cyclin D3 [15]. p57^KIP2^ is an imprinted gene that is conserved between rodents and humans and required for normal development and differentiation [29]. It has been reported that p57^KIP2^ is paternally imprinted and highly expressed in human pancreatic *β*‐cells [30], controling both the self‐renewal and the exit from the cell cycle of pancreatic progenitors during pancreatic development [31]. Targeting p57^KIP2^ promotes adult human *β*‐cell replication [32, 33]. Similarly, downregulation of p57^KIP2^ by FTY720 may promote the differentiated adult human *β*‐cell replication in vivo. S1P signaling plays a key role in adiponectin‐mediated survival of pancreatic *β*‐cells, nutrient uptake and utilization, as well as mitochondrial proliferation [34, 35]. It is apparent that FTY720, a S1P analog, when administered to the diabetic NHPs, acts on an intrinsic pathway that is physiologically important for *β*‐cell survival and regeneration under metabolic stress.

Following the FTY20 treatment, functional *β*‐cell expansion occurs to against the increased insulin resistance in the diabetic NHPs. Our data showed that the FBG and HbA1c still remained at the lowered levels even after withdrawal of FTY720, suggesting that FTY720 may have improved conditions of T2D patients that favors functional *β*‐cell survival. In human T2D patients, *β*‐cell mass decreased due to increased apoptosis [3]. In the studies in *db/db* mice, it was found that the expression of anti‐apoptotic gene products Bcl2 and Bcl‐xL increased in the islets isolated from the *db/db* mice treated with FTY720 [15, 16], indicating newly regenerated pancreatic *β*‐cells are more resistant to apoptotic induction. In addition, Obesity is one of the major risk factors for T2D. It has been reported that FTY720 reverses high‐fat diet‐induced weight gain, insulin resistance and adipose tissue inflammation in C57BL/6 mice [36]. In consistence with this report, the present data showed a mild weight loss in the diabetic NHPs during FTY720 treatment, with the body weight being still slightly lower than the vehicle treated NHPs after withdraw of FTY720 treatment although not significantly different (Figure 6) and insulin resistance is improved (Figure 2), which may also favor the *β*‐cell survival.

T2D is often associated with chronic inflammation that may affect the survival of *β*‐cells [37]. The main mechanisms and molecular signaling of the induction of inflammation in T2D are still unclear. Some evidence showed that stimulated T cells produce cytokines that cooperate with saturated free fatty acids in *β*‐cell destruction in diabetes pathogenesis [38]. FTY720 suppresses the lymphocytes egress from lymph nodes and preferentially traps CD4 + T cells in lymph nodes in MS patients [39]. The T cells trapped in lymph nodes by FTY720 contain the pro‐inflammatory CD4 + TH17 subset that produce IL‐17 and IL‐22, thereby inducing a massive tissue reaction owing to the broad distribution of the IL‐17 and IL‐22 receptors [39]. Interestingly, it was reported that IL‐17 stimulates inducible nitric oxide synthase‐dependent toxicity in mouse *β*‐cells [40]. The present finding that FTY720 treatment in the diabetic NHPs significantly decreased the CD4 + counts (Figure 7) may contribute to the prevention of IL‐17 stimulated *β*‐cell injury. Recently a Nature paper demonstrated that CD8 + T lymphocytes migrate from lymph nodes into pancreas tissue to kill *β*‐cells [11]. Thus, suppression of CD8 + T lymphocytes by FTY720 observed in the diabetic NHPs in the present study may also be attributable to the mechanisms of *β*‐cells protection.

Cardiovascular disease remains one of the leading causes of death in the United States and about 52% T2D patients have a higher risk of mortality at comparable levels of coronary artery disease to those without T2D [41]. Diabetes is a major risk factor for heart failure with preserved ejection fraction (EF), and is highly associated with LV diastolic dysfunction in human [42]. It is expected that anti‐diabetes drug also can improve the cardiovascular function associated with diabetes [43]. Using noninvasive echocardiography, it has been shown that the diabetic NHPs used here are associated with LV diastolic dysfunction similar to that in humans [17]. Indeed, the present data showed that FTY720 treatment increased EF and FS in the diabetic NHPs. Indeed, other research suggests that signaling through the S1P receptor by FTY720 may play a role in the treatment of cardiac microvascular dysfunction in diabetes [44]. It has been proposed that FTY720 might be capable to serve as a potential therapeutic approach for diabetic heart disease through ameliorating cardiac microvascular barrier impairment and pathologic angiogenesis [45]. Although it is notable in clinic that the first dose of FTY720 may cause asymptomatic bradycardia in some patients [46], there was no significant change in heart rate in the diabetic NHPs following FTY720 treatment.

Diabetic nephropathy is the leading cause of end‐stage renal failure worldwide. We previously reported that the diabetic NHP model also accompanied with proteinuria [18], which can be significantly reduced by Losartan, an angiotensin II receptor blocker [22], which is consistent with the report in humans [47]. The present data also demonstrated that FTY720 treatment prevented the progression of proteinuria in the diabetic NHPs. The pathogenesis of diabetic nephropathy is linked very closely to inflammatory cell infiltrations in the kidney [48]. Indeed, it was reported that the number of CD4 + and CD20 + cells correlated with the amount of proteinuria in T2D patient [49]. Although the exact mechanism of this effect remains to be determined, it may attribute to the immunomodulatory effects of FTY720 to decrease T lymphocytes in circulations.

## 5 CONCLUSION

The present study demonstrated that FTY720 sustainedly lowered glycemia, accompanied by rejuvenation of *β*‐cell function, improvement of cardiac function, and reduction of proteinuria in the translational NHP model of diabetes with complication of cardiorenal dysfunctions. The proposed mechanism of action is that FTY720 significantly decreased CD4 + and CD8 + T lymphocytes and increased DC cells leading to prevention of islet *β*‐cell from destruction by autoimmune cells, by which to improve glucose homeostasis. These results strongly suggest that FTY720 as an immunomodulator has a great therapeutic promise for the treatment of T2D with benefit to improve the cardiorenal functions. Because the drug is already approved for treatment of MS by the FDA, it could be tested in clinical trial for its ability to reverse or prevent the progression of T2D.

## CONFLICT OF INTEREST

All the authors were the employees of the participating institutes and declare no conflict of competing interest in this study.

## AUTHORSHIP CONTRIBUTION

YW, XW, WS, YX, GY, and ZM involved in study design, data interpretation and discussion; XW and YX managed experimental execution; AA, LX and MF ran the FACS analysis; KG ran the pharmacokinetic analysis; QL and XL involved bioanalytical analysis of blood samples; YW, XW and ZM wrote the manuscript. All the co‐authors have carefully read and approved the final version of the manuscript for submission to *Journal of Diabetes and its Complications* for publication.

## DECLARATIONS

All experimental protocols and procedures were approved by Crown Biosciences' institutional aminol care and use committee (IACUC) and consented to all participates.

## Supporting information


**Figure S1.** Gait strategy of florescence activating cell sorter (FACS) analysis

## Data Availability

All the experimental data are available upon request.
