# Supplementary material for: Immunomodulator FTY720 improves glucose homeostasis and diabetic complications by rejuvenation of β‐cell function in nonhuman primate model of diabetes
Source: Fundam Clin Pharmacol. 2022 Feb 2;36(4):699–711. doi: 10.1111/fcp.12760 (PMC9546369; doi:10.1111/fcp.12760)
Supplement: Supplementary file 1 — Figure S1. Gait strategy of florescence activating cell sorter (FACS) analysis [file FCP-36-699-s001.docx]

**Immunomodulator FTY720 improves glucose homeostasis and diabetic complications by rejuvenation of β-cell function in nonhuman primate model of diabetes**

**SUPPLEMENTS**

**Analytic method for measurement of plasma concentrations of FTY720**

Plasma concentrations of FTY720 was analyzed by LC/MS-MS method with liquid chromatograph (LC, Agilent 1200) coupled with a tandem quadrupole mass spectrometer (MS, API4000, Singapore) equipped with electro spray ionization (ESI) interface, bin pump (G1312), column oven (G1316C), and CTC PAL auto sampler. The chromatographic separations were achieved using Welch Xtimate C18 column (30 × 2.1 mm; 3µm) at ambient temperature. Lower limit quantification value for FTY720 was 1 ng/ml in the plasma.

The positive ionization modes were employed for the detection of FTY720. The ammonium acetate in water and in 90% MeOH (10 mM) were used as the mobile phase at a flow rate 0.6 mL/min. The main working parameters were set as follows; curtain gas (CUR) 10 L/min; collision gas (CAD) 5 L/min; ion spray voltage (IS) 5500 V; source temperature (TEM) 500^◦^C; and de-clustering potential (DP) 79 V; collision energy (CE) 20 V; entrance potential (EP) 10V; collision cell exit potential (CXP) 6 V; ultra-pure nitrogen gas was used as nebulizer gas (gas1) 60 L/min and heater gas (gas2) 50 L/min. Tolbutamide was selected as internal standard. MS acquisition of FTY720 and IS (tolbutamide) were performed in positive electron spray ionization multiple reaction monitoring (MRM) mode by monitoring the reaction m/z at 308.4 → 255.6 (FTY720), 271.1→ 155.1 (IS), respectively.

**Supplement Figure 1. Gait strategy of florescence activating cell sorter (FACS) analysis**

The blood samples were performed by a flow cytometry with 5 lasers (Fortessa). Total cells were firstly gated then followed by CD56 positive NK and NKT cells and CD3 positive and negative and cell populations. CD4 vs CD8 cell populations were gated from CD3+ cells and CD11c+ DCs were gated from CD3- cells. CD127 and CCR6 expression also been analysis on CD4 and CD8 T cells.
